# Improving Physical Activity Levels in Prevocational Students by Student Participation: Protocol for a Cluster Randomized Controlled Trial

**DOI:** 10.2196/28273

**Published:** 2021-07-28

**Authors:** Huib Van de Kop, Huub Toussaint, Mirka Janssen, Vincent Busch, Arnoud Verhoeff

**Affiliations:** 1 Faculty of Sports and Nutrition Amsterdam University of Applied Sciences Amsterdam Netherlands; 2 Sarphati Amsterdam, Public Health Service (GGD) Amsterdam Netherlands; 3 Faculty of Social and Behavioural Sciences University of Amsterdam Amsterdam Netherlands

**Keywords:** physical activity, participatory, adolescents, protocol, assets, school-based, students, participation, school-age children, teenagers, exercise

## Abstract

**Background:**

A consistent finding in the literature is the decline in physical activity during adolescence, resulting in activity levels below the recommended guidelines. Therefore, promotion of physical activity is recommended specifically for prevocational students.

**Objective:**

This protocol paper describes the background and design of a physical activity promotion intervention study in which prevocational students are invited to participate in the design and implementation of an intervention mix. The intervention is expected to prevent a decline in physical activity in the target group.

**Methods:**

The effectiveness of the intervention was evaluated in a two-group cluster randomized controlled trial with assessments at baseline and 2-year follow-up. A simple randomization was applied, allocating 11 schools to the intervention group and 11 schools to the control group, which followed the regular school curriculum. The research population consisted of 3003 prevocational students, aged 13-15 years. The primary outcome measures were self-reported physical activity levels (screen time, active commuting, and physical activity). As a secondary outcome, direct assessment of physical fitness (leg strength, arm strength, hip flexibility, hand speed, abdominal muscle strength, BMI, and body composition) was included. An intervention-control group comparison was presented for the baseline results. The 2-year interventions began by mapping the assets of the prevocational adolescents of each intervention school using motivational interviewing in the structured interview matrix and the photovoice method. In addition, during focus group sessions, students, school employees, and researchers cocreated and implemented an intervention plan that optimally met the students’ assets and opportunities in the school context. The degree of student participation was evaluated through interviews and questionnaires.

**Results:**

Data collection of the SALVO (stimulating an active lifestyle in prevocational students) study began in October 2015 and was completed in December 2017. Data analyses will be completed in 2021. Baseline comparisons between the intervention and control groups were not significant for age (*P*=.12), screen time behavior (*P*=.53), nonschool active commuting (*P*=.26), total time spent on sports activities (*P*=.32), total physical activities (*P*=.11), hip flexibility (*P*=.22), maximum handgrip (*P*=.47), BMI (*P*=.44), and sum of skinfolds (*P*=.29). Significant differences between the intervention and control groups were found in ethnicity, gender, active commuting to school (*P*=.03), standing broad jump (*P*=.02), bent arm hang (*P*=.01), 10× 5-m sprint (*P*=.01), plate tapping (*P*=.01), sit-ups (*P*=.01), and 20-m shuttle run (*P*=.01).

**Conclusions:**

The SALVO study assesses the effects of a participatory intervention on physical activity and fitness levels in prevocational students. The results of this study may lead to a new understanding of the effectiveness of school-based physical activity interventions when students are invited to participate and cocreate an intervention. This process would provide structured health promotion for future public health.

**Trial Registration:**

ISRCTN Registry ISRCTN35992636; http://www.isrctn.com/ISRCTN35992636

**International Registered Report Identifier (IRRID):**

DERR1-10.2196/28273

## Introduction

### Background

The decline in physical activity levels among young people is an increasing problem. Adolescents in particular show a relapse in sport and exercise participation below the minimum recommended guidelines for physical activity [1-5]. Consequently, the risk of health problems later in life has increased [6,7]. Physical inactivity is a risk factor for chronic diseases, such as cardiovascular disease, cancer, and osteoporosis [8,9]. Therefore, the development and evaluation of interventions with the aim of encouraging adolescents to stay physically active is therefore urgent. Guidelines for adolescents recommend a minimum of 1 hour of moderate-intensity physical activity a day, muscle and bone strengthening exercises three times a week, and avoiding excessive sitting [10].

The school context is potentially an important environment that encourages adolescents to become physically more active [11]. Dutch databases for available school-based interventions contain some *well-described* and *theoretically substantiated* interventions, but little is known about their effectiveness in promoting physical activity [12,13]. Many of these are single risk factor interventions, but the effectiveness of interventions is context dependent, meaning that interventions that might work for senior, general secondary education students cannot immediately be translated to prevocational students [12,14]. Bernaards et al [15] demonstrated that deploying multirisk factor interventions is more effective for prevocational students in terms of increasing physical activity levels [15]. A review of school-based physical activity promotion interventions for prevocational adolescents also indicates that effectiveness increases with an optimal mix of intervention characteristics considering organizational (intracurriculum interventions of short-to-medium duration), personal (tailoring the intervention and empowering students to participate), social (empowering school staff), and content (inclusion of physical activities) determinants [16].

Studies in which students were invited to participate by cocreating an intervention appeared to be potentially more effective [17-19]. Actively involving students using dialog about their perspectives on lifestyle seems to lead to more acceptable and effective interventions [20,21]. It is important to focus on what they enjoy doing and taking into account the possibilities of their local context and characteristics [22]. Therefore, schools aiming to promote healthy lifestyles need to find strategies to involve students to discover their perspectives and empower them for action. This study encourages the full participation of students in the health development process and embraces a *salutogenic* notion of health creation [23,24]. A salutogenic approach seeks the origins of health and focuses on factors that support human health and well-being, rather than factors that cause disease (pathogenesis). People are seen as active and participating subjects, shaping their lives through their action competences [25]. Therefore, it focuses on resources and assets for health and health-promoting processes rather than deficits, risk factors, and disease. As such, a *health asset* can be described as any factor (or resource), which enhances the ability of prevocational students and their social and physical (school) context to maintain and sustain health and well-being and to help reduce health inequities [24]. Resources in a school context are not only a playground, greenery, provision of equipment, and peers of professional school staff but also the capacities and talents of students themselves.

To promote an active lifestyle in prevocational students, it is therefore a challenge to put together an intervention mix that matches specific behavioral determinants (assets) of the students and their environments that support the adoption of a more active lifestyle. This school-based physical activity promotion intervention study, SALVO (stimulating an active lifestyle in prevocational students), aims to evaluate the effectiveness of a physical promotion intervention in prevocational students. This protocol describes the background, design, and baseline results. The results that will be presented include details of the interventions that were developed and baseline characteristics.

### Objectives

The SALVO study is developed to evaluate the effectiveness of a school-based physical activity intervention in improving physical activity behavior in prevocational students. An additional goal of the study is to determine if an intervention is more effective when students participated in the development and implementation process of the intervention. In this paper, we describe the study design and protocol details of the SALVO study.

## Methods

### Objectives and Design

The SALVO study aimed to evaluate the effectiveness of a physical promotion intervention. The intervention was evaluated by a two-group cluster randomized controlled trial (N=3003; 11 intervention schools and 11 control schools) with assessments at baseline (2015) and 2-year follow-up (2016 and 2017). The primary outcome measure was self-reported physical activity as a marker of an active lifestyle. With regard to a secondary outcome, this study examined the effects of interventions on physical fitness measures. The hypotheses tested were as follows:

1. Over 2 years of follow-up, the intervention group had a higher degree of physical activity compared with the control group.
2. Over 2 years of follow-up, the intervention group had a higher physical fitness level compared with the control group.
3. Over 2 years of follow-up, the intervention group with a higher degree of student participation showed a greater intervention effect on the outcome measures compared with the intervention group with a lower degree of student participation.

### Pilot Study

A pilot study aimed to pretest the test battery, and the cocreation and implementation process of the intervention was conducted in students of two prevocational pilot schools aged 12-14 years. The feasibility of the battery measurement during school lessons was evaluated. Furthermore, the usability, comprehension, and acceptability of the interactive methods to involve students in the design and implementation of the intervention were examined.

A valid physical activity questionnaire was filled digitally during mentor hours [26]. A mentor hour is the class time used to acquire skills, such as study skills and social skills. The surveys were conducted in a computer room. Organizing such a room requires preparation in a timetable. The questionnaire was completed under supervision and took 10 to 15 minutes. Together with the instruction, guiding, and use of log-in codes, it was possible to conduct the survey in 20 minutes of class time. The preparation, guidance, and duration required to complete the questionnaire ensured adequate usability, comprehension, and acceptability. Physical performance was tested using the Eurofit test battery during the physical education (PE) lessons [27]. Minor adaptations (class management and use of research assistants) to improve the efficiency of the physical fitness measurement procedures were made.

The pilot study was also used to optimize two action research methods that were deployed to actively engage prevocational students in the SALVO study. Assets were assessed efficiently rather than needs. For this purpose, the structured interview matrix (SIM) and photovoice (PV) were adapted and optimized to fit the interaction with prevocational students in classroom settings. The protocols developed were applied in the pilot schools and adapted iteratively based on the evaluations by critically reflecting on the experiences [28]. The combination of SIM and PV, labeled as triple I, was evaluated as a playful visual method (PV) with an interactive, reflective verbal method (SIM) that was found to work well with the target group of prevocational students.

### SALVO Study: Recruitment of Schools and Students

In accordance with the location of the two Dutch universities collaborating in the SALVO study, 27 prevocational schools located in the provinces of Noord-Holland and Gelderland were invited to participate. If there was a positive response, further information was provided about the design and content during a school visit. Of the 27 schools, five schools indicated that they would not participate because of contented and organizational reasons and wishes. All parents of the students in the second school year received a letter explaining the goals and content of the study and the data collection that went with it. The researchers asked parents a passive form of consent for their child’s participation. Parents and children were given clear instructions on the option to drop out of the study whenever they wanted, without having to give a reason. A total of 6 students of parents who objected were not tested. The study was approved by the ethics committee of the HAN University of Applied Sciences (number ACPO 34.05/16) and retrospectively registered as ISRCTN35992636 in the ISRCTN registry on February 12, 2020 [29].

### Randomization Procedure

**Figure 1:**
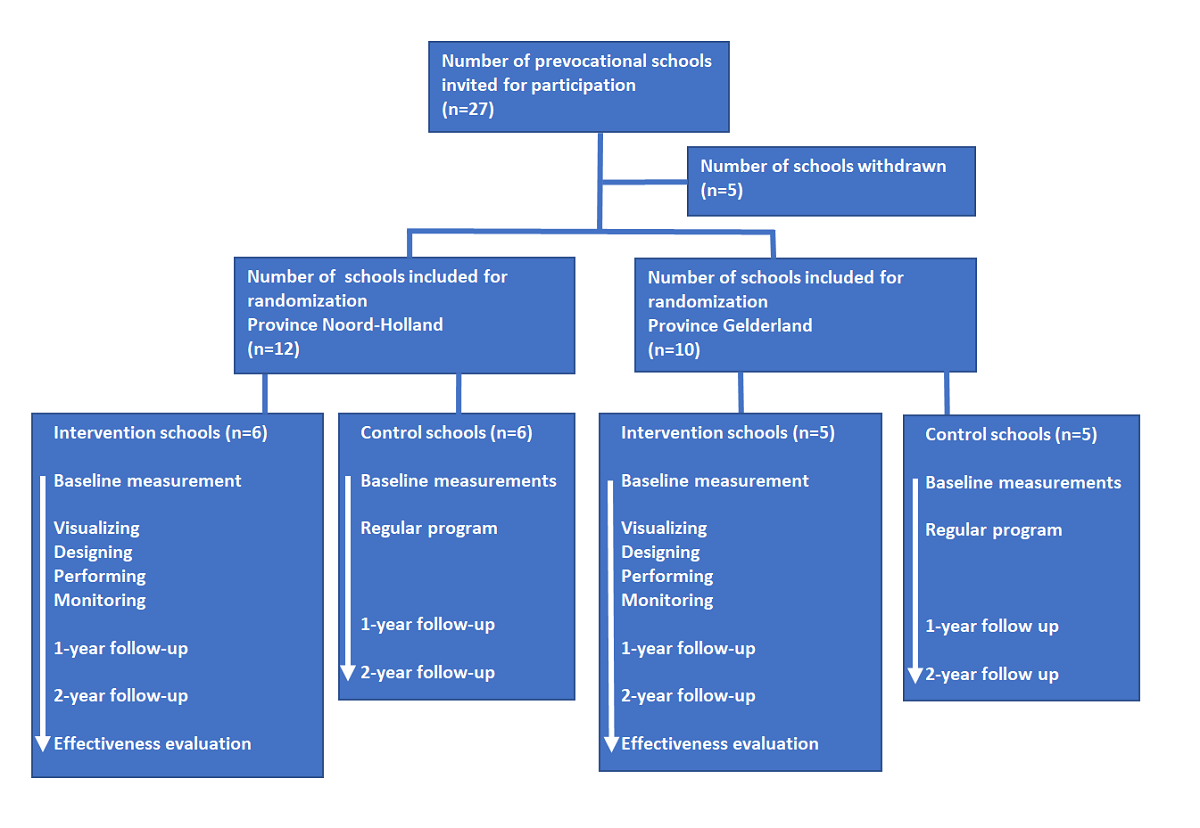
Flowchart of the recruitment and randomization of schools. Timeline for the intervention implementation and evaluation.

A stratified randomization process assigned the 22 participating schools to either the intervention or control group. Schools were stratified according to their location (the district Noord-Holland or Gelderland), with Noord-Holland schools in one stratum and Gelderland schools in the other. A simple randomization was applied, allocating 11 schools to the intervention group and 11 schools to the control group (Figure 1).

### Measurements

The primary outcome measures were physical activity behavior determined using a validated questionnaire and taken digitally during a mentor hour [26]. Textbox 1 presents an overview and description of the variables derived from the questionnaire: screen time, active commuting to school, free time active commuting, time spent in sports, and total physical activity. The physical fitness data were collected using a Eurofit test battery [27]. In two regular PE lessons, the tests were conducted by trained test leaders in accordance with the Eurofit test protocol. Students and test leaders were not blinded, as it is difficult to realize in this kind of research. Prevocational students were instructed on arrival and completed the following components in circuit form (Textbox 1): standing broad jump, bent arm hang, 10× 5-m sprint, sit and reach (sit and reach box), plate tapping, sit-up, and handgrip (Takei hand dynamometer TKK 5401). The next PE class, the 20-m shuttle run, was performed. Anthropometric measures were collected during one of the two classes according to the preferences of the PE teacher. Body weight (Seca robusta 813), body height (Seca 213), and the sum of four skinfolds (Slimguide) were assessed in separate rooms for boys and girls. All measures were taken at baseline and at follow-up after 1 and 2 years.

**Overview and description of the outcome measures included in the study.**

**Physical Activity**

- Screen time (hours/week): hours a week of screen time
- Active commuting school (hours/week): hours a week of walking or cycling to school
- Active commuting other (hours/week): hours a week of walking or cycling to other destinations
- Total time of sport (hours/week): hours a week of sports activities in a club or free time
- Total physical activity (hours/week): hours a week of physical activity in school, club, and free time

**Physical Performance**

- Standing broad jump (cm): explosive leg power
- Bent arm hang (seconds): endurance arm strength
- 10× 5-m sprint (seconds): running speed and agility
- Sit and reach (cm): hip flexibility
- Plate tapping (seconds): arm speed
- Sit-up (numbers/30 seconds): trunk endurance strength
- Handgrip (kg): static arm strength
- 20-m shuttle run: cardiorespiratory endurance

**Anthropometry**

- Sum of skinfolds (mm): sum of four skinfolds
- BMI: body weight/(body height)^2^

### The Intervention

#### Intervention Objectives and Behavioral Goals

The objective of the intervention was to stimulate prevocational adolescents aged 13-15 years to become physically more active and physically more fit. In addition, the objective was to tailor the intervention to the needs and interests of the students themselves by actively involving the students in the cocreation of the intervention. The interventions that resulted from this process were implemented along the possibilities and context of the school. For this reason, tailor-made interventions could differ across schools. The control group went through a regular school curriculum without the development and implementation phases of the intervention. Table 1 presents an overview of the intervention characteristics for each school. A description of one of the interventions is included as an example in Textbox 2.

**Table 1:** Estimation of intervention characteristics in the intervention schools.

Intervention school		1	2	3	4	5	6	7	8	9	10	11
Physical activities		✓^a^	✓	✓	✓	✓	✓	✓	✓	✓	✓	✓
**Curriculum**												
	Intra				✓	✓	✓			✓		
	Extra	✓	✓	✓	✓		✓	✓	✓		✓	✓
School staff participation		✓	✓	✓	✓	✓	✓	✓	✓	✓	✓	✓
School management participation										✓		✓
Tailored intervention			✓	✓	✓	✓		✓				
**Student participation**												
	Low	✓					✓	✓				✓
	Moderate		✓	✓		✓			✓	✓	✓	
	High				✓							

^a^The intervention of the school includes the intervention characteristic.

**Example of an intervention cocreated by students and teachers.**

School A offers prevocational courses aimed at the agricultural sector. It recently has moved into a new building. From the assets determination, students indicated that the new schoolyard is empty and boring. The students' wish is to use the school playground during breaks to play and exercise. After focus group sessions, the plan is to make small sports equipment available from the gym during school breaks. Students coordinate the distribution and cleaning up of the equipment. The regional Sports Service Centre supports by providing larger equipment that is borrowed and replaced on a monthly basis (soccer goals and basketball installation). One of the handicraft teachers decides to build the soccer goals during the lesson by the students themselves so that they can be used permanently. The schoolyard has transformed into a daily useful playground for the students. The success of the solution did lead to some nuisance complaints from teaching teachers about the sound produced by the playing students.

#### Theoretical Background and Determinants Addressed

Within the salutogenic framework, health was seen as a process in which people are always in some regard healthy and independent of existing distress and diseases [30]. It focuses on resources and assets for health and health-promoting processes, rather than deficits, risk factors, and disease. The values and principles of the asset model emphasize the need to strengthen local communities [24]. Morgan [24] defined health assets as any factor or resource that enhances the ability of individuals, groups, communities, populations, social systems, and institutions to maintain health and well-being and help to reduce health inequities. These assets could work at the level of the individual, group, or population as protective or promoting factors to buffer against life’s stresses. The SALVO study embraced the model through asset mapping to promote community empowerment. It created supportive (healthy) environments by helping to identify the key assets that generate living and working conditions that are safe, stimulating, satisfying, and enjoyable.

### General Intervention Framework

In line with the salutogenic framework, the SALVO study focused on the strengths and resources that strengthen the ability of prevocational students to become physically more active. Therefore, the process of intervention development and implementation was conducted in the intervention schools and followed several phases to respond to students’ personal motives (Table 2). During the start-up phase (visualizing), the behavioral determinants of students were assessed based on asset mapping. The positive aspects of students are listed and ranked on the basis of SIM, PV, and focus group sessions. The Morgan study [24] showed that asset mapping ensures more involvement and health control for participants. Co-designing the intervention with students ensured a better connection with the experienced world (context) of prevocational students, so that a more active lifestyle could be developed on the basis of intrinsic motivation [24]. Asset mapping was performed with a subsample of prevocational adolescents (one class) in each intervention school. Motivational interviewing in the SIM and PV methods was used, taking into account the application of relevant quality procedures [31]. The SIM examined what students thought of an active lifestyle and how they knew how to follow up on this lifestyle [32]. In the PV method, students used photos to present opportunities for an active lifestyle in the school environment and their own neighborhood. These photos were then presented to each other with an oral explanation [33,34]. All conversations were supervised by trained research assistants and were voice recorded. Both students and school teachers actively participated in this process of asset mapping. The goal of asset mapping was to identify which resources may contribute to the more active lifestyle of students. All SIM and PV recordings were analyzed using the standards for qualitative research [35]. The assets were mapped on the basis of four factors that influence the physical activity behavior of prevocational students: social environment, physical environment, personal skills, and passions and interests. The results were interpreted by researchers and, in the next phase, the students were checked to what extent this interpretation matched their conception. During the design phase in each intervention school, two focus group sessions were held with a subsample of 4-6 students, researchers, members of the teaching staff, and school board. The goal was to create an intervention plan. In the triangulation process, the drivers of behavior (assets) were matched with an inventory of databases containing the existing well-described and substantiated interventions for this target group and the opportunities for implementation of an intervention provided by the school. During the focus group sessions, students were encouraged to advise and co-decide on the development of the intervention [32]. Extra focus group sessions were held if external stakeholders, such as providers of sports activities, played a role in conducting the intervention. In the implementation phase (performing), the students and teachers jointly implemented the intervention mix. The researchers monitored the implementation process through regular conversations with all the stakeholders. The final phase—the evaluation phase (monitoring)—was intended to evaluate the intervention mix. In this phase, the intervention was evaluated based on the degree of alignment with the wishes and needs of the students. The findings arising from this process evaluation were used to initiate a new process of cocreation. Students and employees actively participated in this process.

**Table 2:** Summary of the goals, methods used, and results of the intervention design and implementation phases of the SALVO (stimulating an active lifestyle in prevocational students) study.

Phase and goal		Method	Result
**Visualizing**			
	Students’ asset mapping	Structured interview matrix Photovoice Qualitative analysis	Social environment Physical environment Motor skills Passions and interests
**Designing**			
	Cocreation intervention	Focus group sessions	Intervention plan
**Performing**			
	Intervention implementation	N/A^a^	Intervention activities
**Monitoring**			
	Evaluation of intervention mix	Focus group sessions	Intervention adjustments

^a^N/A: not applicable.

### Sample Size

The sample size was based on a power analysis using the results of a previous Dutch study, in which an increase from 41% to 55% of norm actives was reported in 17,891 prevocational students [15]. Assuming a similar effect with a power of 0.90 and an α of .05, a required number of 536 students were divided into two groups. Taking into account the cluster effect, the sample size was calculated for 901 students with an intracluster correlation of 10%.

### Procedure of the Process Evaluation

During the implementation phase of the intervention, consultation moments between the researchers and teachers were planned on a regular basis. The purpose of these consultations was to monitor the implementation and, if necessary, adjustment of this process. After the intervention period, interviews were conducted with the students and teachers for each intervention school. Surveys determined the degree of student participation during the design and implementation phases. The degree of participation was expressed ordinally from weak (to inform) to moderate (to think along) and strong (to co-decide) [36].

### Statistical Analysis

The effectiveness of the SALVO intervention was analyzed using a multivariable analysis. Therefore, generalized estimating equations (GEEs) were used to evaluate the intervention that explains the variability between the intervention and control groups on the outcome variables of physical activity and physical fitness. GEEs are chosen to account for the possible intragroup correlation, and an exchangeable correlation structure was assumed for these analyses. The coefficient of interest in this analysis is the regression coefficient of the interaction between the group (intervention or control) and time [37]. One model considers the explanatory variables over time, including baseline values of the outcome variable, gender and ethnicity, the group (intervention or control), time, and the interaction between group and time. The second model presents a sensitivity analysis that includes intervention schools with moderate to strong levels of participation of students or intervention schools with the lowest level of student participation. The results of GEE analyses are expressed as the β coefficient of the interaction between group and time, with corresponding 95% CI and associated *P* values. In this design and protocol paper, the results of the baseline comparison between the intervention and control groups for physical activity and physical fitness data are presented. In addition, the distributions of gender, ethnicity, and age are presented. A two-tailed independent *t* test and chi-square test were performed for continuous and nominal variables, respectively. *A priori*, the criterion for statistical significance, was set at *P*<.05. All analyses were performed using the SPSS software (version 26; IBM Corp).

## Results

### Demographics

The total study population of 3003 students consists of 1457 girls (48.52%) and 1546 boys (51.48%) aged 13.8 years (SD 0.5). The students mainly had a Dutch background (1754/2640, 66.44%). The reported countries of origin of the parents of children with a migration background were Morocco, Turkey, Suriname, the Netherlands Antilles, Poland, Iraq, and Somalia. A total of 42.16% (1266/3003) students lived in the province of Gelderland. A total of 57.84% (1737/3003) students lived in the province of Noord-Holland.

Table 3 provides the baseline results for the distribution of gender and ethnicity in the intervention and control groups. Boys were overrepresented in the control group (*P*<.001), whereas students with a migration background were overrepresented in the intervention group (*P*<.001). The mean age of the population in the control and intervention groups was similar (*P*=.12; Table 4).

**Table 3:** Baseline demographic and gender comparisons between the intervention and control groups.

Characteristic		Control group, n/N (%)	Intervention group, n/N (%)	*P* value	
**Gender**					<.001
	Girls	725/1611 (45)	732/1392 (52.58)		
	Boys	886/1611 (54.99)	660/1392 (47.41)		
**Ethnicity (n=2640)**					<.001
	Domestic	1042/1455 (71.62)	712/1185 (60.08)		
	Immigrant	413/1455 (28.38)	473/1185 (39.92)		

**Table 4:** Baseline physical activity and physical fitness comparison between the intervention and control groups.

Baseline characteristic			Girls (N=725)				Boys (N=886)		
			Students, n (%)		Values, mean (SD)		Students, n (%)		Values, mean (SD)
**Control**									
	Age (years)	725 (100)		13.8 (0.5)		886 (100)		13.8 (0.5)	
	Screen time (hours/week)	493 (68)		52.1 (0.5)		566 (63.8)		55.1 (17.3)	
	Active commuting school (hours/week)	487 (67.2)		1.2 (0.5)		561 (63.3)		1.31 (1.0)	
	Active commuting other (hours/week)	248 (34.2)		0.5 (0.5)		229 (25.9)		0.5 (0.5)	
	Total time of sport (hours/week)	385 (53.1)		4.3 (0.5)		456 (51.5)		5.1 (2.4)	
	Total physical activity (hours/week)	479 (66.1)		5.0 (0.5)		559 (63.1)		5.6 (3.1)	
	Standing broad jump (cm)	643 (88.7)		139.5 (22.7)		785 (88.6)		158.0 (25.8)	
	Bent arm hang (Ln^a^)	642 (88.6)		1.3 (1.5)		787 (88.8)		2.3 (1.3)	
	10× 5-m sprint (seconds)	634 (87.5)		21.5 (1.9)		773 (87.3)		19.8 (1.7)	
	Sit and reach (cm)	651 (89.8)		27.1 (8.3)		788 (88.9)		20.5 (7.3)	
	Plate tapping (seconds)	653 (90.0)		12.6 (1.8)		790 (89.1)		12.5 (1.7)	
	Sit-up (numbers/30 seconds)	644 (88.9)		18 (4)		784 (88.5)		22 (4)	
	Handgrip (kg)	656 (90.5)		29.4 (5.4)		790 (89.2)		32.6 (7.5)	
	20-m shuttle run (score)	511 (70.5)		5.9 (2.0)		649 (73.3)		7.8 (2.2)	
	Sum of skinfolds (Ln)	635 (87.6)		3.0 (0.2)		783 (88.4)		3.0 (0.2)	
	BMI (Ln)	634 (87.4)		4.0 (0.4)		779 (87.9)		3.5 (0.5)	
**Intervention**									
	Age (years)	732 (100)		13.8 (0.5)		660 (100)		13.8 (0.5)	
	Screen time (hours/week)	515 (70.4)		52.1 (18.2)		464 (70.3)		56.6 (18.6)	
	Active commuting school (hours/week)	514 (70.2)		1.4 (1.0)		461 (69.9)		1.3 (1.0)	
	Active commuting other (hours/week)	225 (30.7)		0.4 (0.5)		210 (31.8)		0.6 (0.6)	
	Total time of sport (hours/week)	359 (49.0)		4.1 (2.4)		364 (55.2)		5,1 (2.5)	
	Total physical activity (hours/week)	482 (65.9)		4.8 (3.1)		453 (68.6)		5.7 (3.3)	
	Standing broad jump (cm)	624 (85.2)		139.7 (21.9)		597 (90.5)		155.6 (24.1)	
	Bent arm hang (Ln)	612 (83.6)		1.1 (1.5)		575 (87.1)		2.0 (1.5)	
	10× 5-m sprint (seconds)	620 (84.7)		21.9 (2.2)		590 (89.4)		20.5 (2.0)	
	Sit and reach (cm)	630 (86.1)		26.0 (8.5)		603 (91.4)		20.0 (7.5)	
	Plate tapping (seconds)	630 (86.1)		13.0 (2.0)		604 (91.5)		12.8 (1.9)	
	Sit-up (numbers/30 seconds)	626 (85.5)		17 (4)		603 (91.4)		21 (4)	
	Handgrip (kg)	633 (86.5)		29.4 (5.6)		606 (91.8)		32.5 (8.2)	
	20-m shuttle run (score)	387 (52.9)		5.3 (2.3)		401 (60.8)		7.3 (2.7)	
	Sum of skinfolds (Ln)	613 (84.7)		3.0 (0.2)		596 (90.3)		3.0 (0.2)	
	BMI (Ln)	620 (84.7)		3.9 (0.4)		596 (90.3)		3.5 (0.5)	

^a^Ln: log-linear transformed data.

### Physical Activity

From the total student population (N=3003), 2286 (76.12%) students participated in the baseline measurements (Table 4). During the week, 28.39% (560/1972) of the total population reported being physically active for at least seven hours. The mean total physical activity level of 1983 students was 5.3 hours a week (SD 3.2). Sports activities contribute the most, with 4.7 hours a week (SD 2.5). The students spend approximately 1.5 hours a week walking or cycling to school. Screen time use among 2038 students is 53.9 hours a week (SD 18.5). A comparison between 1054 intervention students and 1232 controls did not show significant differences in screen time behavior (*P*=.53), active commuting other than going to school (*P*=.26), total time spent on sports activities (*P*=.32), and total time spent on physical activity (*P*=.11). Intervention students spent more time on active commuting to school than did the controls (*P*=.03).

### Physical Performance and Anthropometry

Physical fitness was assessed in 2566 students (1168 intervention vs 1398 controls). Table 4 presents the baseline gender-specific results of the outcome measures in the intervention and control groups. The variables that were not normally distributed were log-linear transformed (bent arm hang, BMI, and sum skinfolds). The mean hip flexibility (*P*=.22) and maximum hand grip strength (*P*=.47) were comparable between the intervention and control groups. Other physical performance parameters were found to be significantly better for students in the control group. Baseline outcome values for body composition (*P*=.29) and body mass (*P*=.44) were similar between the intervention and control groups.

## Discussion

### Principal Findings

The aim of the SALVO study is to promote an active lifestyle in prevocational students by allowing students to participate in intervention development and implementation. To this end, students have been invited to participate in dialog with peers and school staff. It is expected that the alignment of students’ assets and interventions will lead to a meaningful basis for a sustainable active lifestyle of students.

Most of the prevocational students in this study did not meet the minimum guidelines for healthy exercise and showed a high degree of screen use. The physical fitness of boys exceeds that of girls, except for hip flexibility.

Normative values for physical fitness were published in a recent review of 2,779,165 adolescents from 30 European countries [38]. Compared with the normative centile scores of their peers, the students aged 14 years in this study achieved relatively low scores on standing broad jump (P10-30), plate tapping (P30-40), and sit-ups (30-40). In addition, students who can barely hang from bent arms are overrepresented. In contrast, hip flexibility (P60) and the shuttle run test scores (P60-80) were relatively better developed in boys and girls. Hand grip scores of girls (P70) were comparatively better than those of boys (P40). The 10× 5-m shuttle run agility test scores were normative (P50-60). Finally, students with high BMI and sum or skinfold values are overrepresented.

The mean total physical activity level found in this study of 5.3 hours (SD 3.2) a week is less than the 18 hours a week reported by the Dutch National Institute for Public Health and the Environment [5]. A possible reason for this is the difference in the questionnaires used. In addition, the study population in this study is specifically aimed at prevocational secondary education students instead of all young people between the ages of 12 and 19 years. Physical activity such as sports activities and cycling are the main physical activities that contribute to the total physical activity of this population. This is in line with data reported by the Dutch National Institute for Public Health and the Environment [5]. The prevalence of students that meet the physical activity guidelines for Dutch adolescents found in this study is 28.39% (560/1972), which corresponds to the levels of 28% reported by the Dutch Health Council [10]. The prevalence of insufficient physical activity of 71.6% (1412/1972) is approximately 7% less than the internationally reported percentage of 78.2% for high-income Western countries [39].

The participatory approach is sparsely used in research when evaluating active lifestyle interventions among prevocational secondary education students [31,40-44]. This is surprising, since the involvement of students in developing an intervention is considered an effective intervention characteristic [16]. This research will provide insight into the effects of such a participatory approach on physical activity and fitness among prevocational students.

### Strengths and Limitations

The strength of this study is that the design process of the intervention is based on students’ assets, existing and theoretically well-described interventions for this target group, and the opportunities and possibilities offered by the school context. This triangulation process unfolds in focus group sessions between different stakeholders, such as school management, researchers, and staff. It provides valuable practical experiences for every school that would like to support students in developing an active lifestyle. Another strength of the research is the size of the number of participants and the experimental design. Evidence-based practice and practice-based evidence meet in this study.

Considering that there are few studies that have rigorously investigated the participation of students in intervention development, the SALVO study will provide needed insight into the promotion of physical activity in a school context. The results of this study could help in creating more refined and successful school-based physical activity interventions in the future.

## Abbreviations

GEE: generalized estimating equation
PE: physical education
PV: photovoice
SALVO: stimulating an active lifestyle in prevocational students
SIM: structured interview matrix
